# Colorimetric and Fluorescent Sensing of Copper Ions in Water through o-Phenylenediamine-Derived Carbon Dots

**DOI:** 10.3390/s23063029

**Published:** 2023-03-10

**Authors:** Roberto Pizzoferrato, Ramanand Bisauriya, Simonetta Antonaroli, Marcello Cabibbo, Artur J. Moro

**Affiliations:** 1 Department of Industrial Engineering, University of Rome Tor Vergata, 00133 Rome, Italy; pizzoferrato@uniroma2.it; 2 Department of Chemical Sciences and Technology, University of Rome Tor Vergata, 00133 Rome, Italy; 3 Department of Industrial Engineering and Mathematical Sciences (DIISM), Università Politecnica Delle Marche, 60131 Ancona, Italy; 4 LAQV-REQUIMTE, Departamento de Química, CQFB, Universidade Nova de Lisboa, 2829-516 Caparica, Portugal

**Keywords:** carbon dots, o-Phenylenediamine, optical sensing, copper, colorimetric, fluorescence, agarose hydrogel

## Abstract

Fluorescent nitrogen and sulfur co-doped carbon dots (NSCDs) were synthesized using a simple one-step hydrothermal method starting from o-phenylenediamine (OPD) and ammonium sulfide. The prepared NSCDs presented a selective dual optical response to Cu(II) in water through the arising of an absorption band at 660 nm and simultaneous fluorescence enhancement at 564 nm. The first effect was attributed to formation of cuprammonium complexes through coordination with amino functional groups of NSCDs. Alternatively, fluorescence enhancement can be explained by the oxidation of residual OPD bound to NSCDs. Both absorbance and fluorescence showed a linear increase with an increase of Cu(II) concentration in the range 1–100 µM, with the lowest detection limit of 100 nM and 1 µM, respectively. NSCDs were successfully incorporated in a hydrogel agarose matrix for easier handling and application to sensing. The formation of cuprammonium complexes was strongly hampered in an agarose matrix while oxidation of OPD was still effective. As a result, color variations could be perceived both under white light and UV light for concentrations as low as 10 µM. Since these color changes were similarly perceived in tap and lake water samples, the present method could be a promising candidate for simple, cost-effective visual monitoring of copper onsite.

## 1. Introduction

The continuous accumulation and non-biodegradability of heavy metals (HMs) in the hydro- and biosphere cause a wide variety of environmental problems and diseases [1,2] both owing to their natural presence and to growing use in man-made activities, with a consequently increasing presence in industrial effluents [3,4]. Thus, rising levels of HMs in surface and ground water have been reported in recent years in both developed and undeveloped countries. One such heavy metal is copper (Cu(II)), which is an essential microelement for several biological processes not only in humans, such as metabolism, growth, and immune system development [5], but also in plants where it combines with enzymes to perform many nutritional functions [6]. Despite this beneficial role, it is commonly known to accumulate in fish (specifically in gills), where it causes mortality through respiratory disruption [1,2,7], bacteria, and viruses [8]. In humans, long-term exposure to Cu(II) can give rise to liver and kidney damage, gastrointestinal disturbance, and numerous neurodegenerative syndromes, such as Alzheimer’s, Wilson’s, and Parkinson’s diseases [9,10].

Based on the risks highlighted above, it is necessary to develop highly selective, sensitive, and efficient methods that can detect low concentrations of Cu(II) in the environment such as the standards set for drinking water by the US Environmental Protection Agency (EPA) of 1.3 µg/mL (20 µM) and the World Health Organization (WHO) of 2.0 µg/mL (32 µM) [11]. A number of analytical approaches, such as electrochemical [12], atomic fluorescence spectrometry [13], inductively coupled plasma mass spectrometry [14] and atomic absorption/emission spectroscopy [15] have been utilized for quantitative assaying of heavy metal ions in aqueous solution with high sensitivity and accuracy. However, these methods imply complicated and expensive instruments, chemical pre-treatments, long time assay, highly-skilled users, and are non-portable, thus preventing rapid, easy and low-cost on-site detection [16].

Fluorescence spectroscopy is an effective optical detection technique that covers all the requirements such as high selectivity and sensitivity, a wide linear dynamic range, easy operation, possibility of remote monitoring, and a promising capability for rapid real-time monitoring [17,18] of heavy metals such as Cu(II). It is generally based on conventional organic reactants or dyes [19]. More recently, nano-sensor materials, such as semiconductor quantum dots [20], have expanded the range of analytes and possibilities of fluorescence spectroscopy. However, organic compounds are limited by complicated synthesis methods and photobleaching [21], while semiconductor quantum dots have major issues such as high toxicity, intrinsic blinking, chemical instability and insolubility in aqueous solution [22].

Among the organic reactants, o-Phenylenediamine (OPD) has recently been investigated for optical sensing of copper and silver ions since these two metal species oxidize OPD to 2,3-diaminophenazine (OPD_ox_), and the latter gives a fluorescent emission at 564 nm [23,24,25,26]. This effect was also exploited in combination with carbon dots (CDs), a new emerging class of fluorescent nanomaterials, which act as catalyzers and makes the oxidation reaction quicker and more simple [27,28,29,30]. Based on this effect, sensing is generally accomplished through fluorescence enhancement occurring in the presence of metal ions.

In fact, CDs have received extensive attention from researchers in multiple disciplines due to their novel mix of properties, such as low production cost, solubility in water, low cytotoxicity, resistance to photobleaching, robust chemical inertness, good biocompatibility, and abundance of functional groups, compared with the aforementioned conventional probes [11,31]. They are promising candidates in many fields of application, but attracted researchers the most by demonstrating a potential for fluorescent/colorimetric sensing of HMs ions such as Hg(II), Pb(II), Cu(II), Cr(VI), Cr(III), Fe(III) [32,33,34,35,36,37,38], and other analytes [39]. However, one of the limiting factors of sensing with CDs is the need of mixing the targeted aqueous sample with the liquid sensing solution. This makes the method more complicated and less reproducible than desired and hinders on-site detection. To prevent these drawbacks, much effort has been made to immobilize the CDs into solid-state or hydrogel matrices [31,40,41,42,43].

We have previously synthesized nitrogen and sulfur co-doped CDs (NSCDs) by using OPD and ammonium sulfate as the two precursors in a hydrothermal method [44]. Both the starting compounds added nitrogen-containing functional groups to the carbon structure, thus enabling the formation of cuprammonium CD-complexes when the NSCDs interact with Cu(II) ions. Since cuprammonium complexes present a distinct optical absorption band at 660 nm, colorimetric sensing of copper in water was achieved through variation of optical absorbance with a limit of detection (LOD) of 100 nM. We also noticed that the hydrothermal synthesis endowed the NSCDs with the typical fluorescent emission of OPD_ox_ at 564 nm, possibly owing to some residual OPD and OPD_ox_ molecules bound to the carbon structure. Here we investigate how this fluorescent emission is enhanced by the presence of copper ions, due to further oxidation, simultaneously with the rising of the absorption band at 660 nm. In addition, we incorporated the NSCDs in an agarose gel matrix and studied how the two optical effects changed, and how sensing of copper could be more easily be achieved by simple visual observation, even in real water samples. The overall behavior of NSCDs thus makes them a possible system for dual colorimetric-fluorescent sensing system for copper ions in water.

## 2. Materials and Experimental Procedure:

### 2.1. Materials

All chemicals used throughout the experimental procedure were purchased from Merck Sigma Aldrich (Merk Life Science S.r.l., Milano, Italy) and all are of analytical reagent grade and used as received. O-phenylenediamine was used as a carbon source and ammonium sulfate as a source of N and S. The sensitivity and selectivity tests were performed on the following heavy metal salts: HgCl_2_, K_2_Cr_2_O_7_, Pb(NO_3_)_2_, Fe(NO_3_)_3_⋅9H_2_O, CuCl_2_⋅2H_2_O, Cd(NO_3_)_2_⋅4H_2_O, CoCl_2_⋅6H_2_O, NiCl_2_⋅6H_2_O, NaAsO_2_, and AgNO_3_. Milli-Q de-ionized (DI) water ((18.25 MΩ cm, Millipore, Milford, MA, USA) was used to prepare all solutions. The pH was varied with HNO_3_ (37%) and NaOH (1 M solution) for pH studies.

### 2.2. Synthesis of Carbon Dots

The fluorescent carbon dots were synthesized by following a procedure previously reported [44] based on a typical hydrothermal method. Briefly, the precursors o-phenylenediamine and ammonium sulphate solutions (prepared in Ethanol and DI water, respectively) were mixed and transferred to a Teflon-lined stainless-steel autoclave and heated at 220 °C for 6 h. The resulting product was filtered with a 0.22 µm polyethersulphone membrane and dialyzed in DI water through a dialysis bag with a cut-off of 2 kDa (Sigma-Aldrich Merk Life Science S.r.l., Milano, Italy). Finally, the pH was adjusted to 9.5 by using NaOH and HNO_3_, since this value gave the highest sensitivity and selectivity.

### 2.3. Agarose Hydrogel Film Preparation

A typical procedure was adopted to prepare the agarose hydrogels, in which 2.5% (*w*/*v*) was set by dissolving 0.25 g of agarose in 10 mL of DI water at a pH adjusted to 9.5, followed by heating at 60 °C for 20 min. The hydrogel film casting was made by pouring the 2 mL of above solution into 2 × 2 cm^2^ plastic cubes and leaving these to dry for 2 h at room temperature in atmospheric conditions. Finally, a square hydrogel film of average thickness of ~2 mm was easily removed.

### 2.4. Carbon Dots incorporated Agarose (Aga-NSCDs) Hydrogel Film Preparation

The *Aga-NSCDs* hydrogel film preparation was the same as that for pure agarose with the only difference that the NSCDs-sensing solution was used in place of DI water. The solution was then heated at 60 °C for 20 min to complete the solubility of agarose, and film casting was achieved by following the same protocol as used in the preparation of the pure agarose hydrogel film.

### 2.5. Instrumentation for Characterization and Sensitivity Measurements

In order to investigate the presence of functional groups, Fourier-transform infrared spectra (FTIR) were recorded through a spectrometer Spectrum 100 FTIR, (Perkin Elmer Italia Spa, Milano, Italy) in the range between 400 and 4000 cm^−1^. The NSCD water samples were dried in nitrogen atmosphere for 24 h at 50 °C and analyzed in KBr cells.

Dynamic light-scattering (DLS) measurements were performed on a Nanoparticle Analyzer SZ-100 (Horiba Scientific, Kyoto, Japan), in 3 mL (10 mm × 10 mm, width × depth) fully transparent PMMA cells. Prior to each measurement, the samples were filtered using a 0.46 µm polystyrene membrane disc filter. Zeta-potential (ζ-potential) experiments were performed on the same instrument but using specific microelectronic capillary cuvettes (100 µL, Horiba Scientific, Kyoto, Japan). Calculation of ζ-potential for all samples was made using the Smoluchowski model. All measurements from both DLS and ζ -potential were performed in triplicate. Transmission Electron Microscopy (TEM) inspections were performed by using a Philips^TM^ CM-20^®^ microscope (Philips, Amsterdam, the Netherlands) operating at 200 kV and equipped with a liquid-nitrogen stage to keep the samples cooled. The samples were prepared by drying a drop of NSCD solution on carbon film and fixing the carbon particles on the substrate with infrared radiation for a time ranging from 30 to 120 s. Statistical evaluation of NSCD mean equivalent diameter was carried out by considering at least 150 features and using an image analysis software (Leica^TM^ Image Pro Plus^®^ 4.5.1 Materials Pro).

A Cary 50 spectrophotometer (Varian Inc., Palo Alto, CA, USA) was used to acquire UV–Vis absorption spectra. Fluorescence emission spectra were recorded by using a laboratory set-up equipped with a discharge Hg-Xe lamp (Oriel Instruments, Stratford, CT, USA) and a 25-cm monochromator (Photon Technology International, Inc., Birmingham, NJ, USA) for the excitation light. Fluorescence was dispersed by a 25-cm monochromator (Oriel Cornerstone 260, Oriel Instruments, Stratford, CT, USA) and revealed through a photomultiplier (R3896, Hamamatsu Photonics Italia S.r.l., Milano, Italy). Liquid samples were held in fused silica cuvettes, using an optical path of 10 mm and a typical 90-degree geometry and taking care to minimize the inner filter effect. Dynamic fluorescence measurements were performed with a KOALA-ISS fluorometer (ISS, Champaign, IL, USA), using phase shift and demodulation techniques. The excitation source (450 nm) was a laser diode; emission was collected through a 490 WG cut-off filter to avoid scattered light. The data were fitted according to a single exponential decay time.

### 2.6. Determination of Cu(II) Ions in Liquid and Solid State

The titrations for sensitivity tests in liquid was performed as follows. First, 1 mL of DI water was added to 1 mL of NSCDs-sensing solution and gently stirred for 10 s to prepare the blank (reference) solution, which was used against the typical sensing experiment in the presence of a heavy metal (HM). This was performed by adding 1 mL of the specific HM salt solution in DI water at the prearranged ion concentration to 1 mL of NSCDs-sensing solution and gently stirring for 10 s. The sensing measurement with the solid-state agarose matrix for Cu(II) determination was made against the reference by dipping the *Aga-NSCDs* films in the excess DI water (for reference) and in the prescribed Cu(II) ion concentration solution, respectively. The real water sensing was performed by the same procedure as for DI with the only difference being that the real water samples were spiked with concentrated salt solutions to achieve the prescribed HM assay concentration. All the spectra were recorded 10 min after mixing the water samples with the NSCD sensing solution (in the liquid sample sensing) and 2 h after dipping the hydrogel film in the respective HMs solution (in solid-state sensing).

## 3. Results

### 3.1. Morphological and Structural Characterization

**Figure 1:**
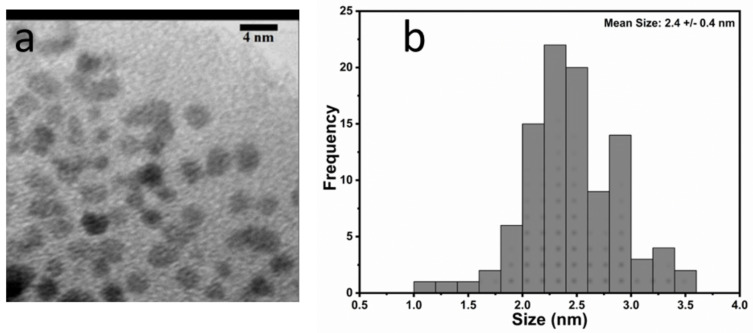
(**a**) BF-TEM micrograph and (**b**) the statistical size distribution of a typical population of NSCDs.

**Figure 2:**
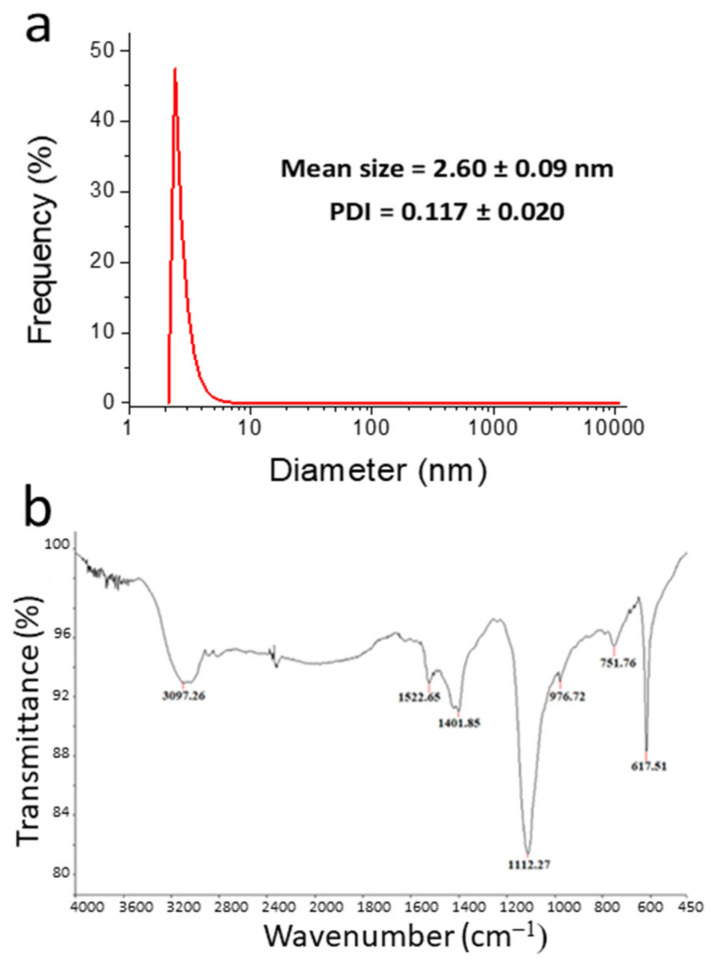
(**a**) DLS measurement and (**b**) FTIR spectrum of NSCDs.

A representative bright-field (BF) TEM micrograph of pure NSCDs is shown in Figure 1a, while the relative statistical size distribution is reported in Figure 1b. As generally observed for similar hydrothermal preparations, it appeared that the particles presented quite a narrow size distribution. In the present case, we reported an average diameter of 2.4 ± 0.4 nm. The DLS measurements (see Figure 2a) substantially confirmed that this size and, importantly, the monodispersity of NSCDs still held in water dispersion. The ζ-potential of NSCDs is close to 0 (see Figure S1a in Supplementary Materials) as expected since the amines that are on the surface should be close to neutral in the pH conditions of the sensing solution.

The infrared spectrum of NSCDs is shown in Figure 2b. Most of the observed peaks can be attributed to the different functional groups produced by the synthesis procedure; specifically, the peaks at 1112 cm^−1^ and 1401 cm^−1^ can be assigned to S = O bonds and C-S bonds, respectively, thus confirming the presence of sulfur [45]. In addition, the peak at 1523 cm^−1^ is due to C = N, while that at 752 cm^−1^ comes from N-H wagging [46,47]. Finally, the sharp and intense peak at 618 cm^−1^ might be assigned to N-C bending of phenyl nitrogen groups [48].

### 3.2. Spectroscopic Characterization

**Figure 3:**
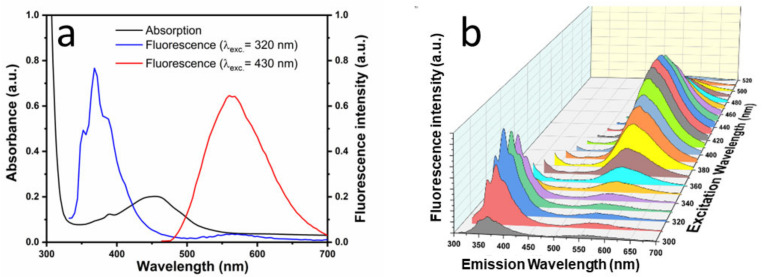
(**a**) UV-Vis absorption spectrum (black curve) and fluorescence spectra excited at λ_exc_ = 320 nm (blue) and λ_exc_ = 430 nm (red) of NSCDs; (**b**) emission spectra at different excitation wavelengths.

Figure 3a displays the absorbance and fluorescence spectra of the NSCD solution. The UV-Vis absorption spectrum (black line) shows a steep increase in absorbance towards the short-wavelength side, which is generally assigned to the tail of the high-energy π-π* optical transitions of aromatic C = C bonds in isolated sp^2^-carbon domains in the highly defective graphene-like carbon lattice [49]. Similarly, the n-π* transitions of nitrogen- or oxygen-containing functional groups of NSCDs can give rise to the small peaks that emerge from the tail in the range 360–380 nm. However, we believe that the distinct broad absorption band around 460 nm is not directly related to NSCDs but can rather be attributed to the presence of residual oxidized o-Phenylenediamine (OPD_ox_) adsorbed on the surface of CDs through electrostatic interaction [28,30]. This effect will be discussed further in Section 3.3.

As regards the photoluminescence (PL) spectra, we note that an intense structure at 370 nm is produced by UV excitation. It can be explained by the intrinsic UV/blue emission of the isolated sp^2^ carbon domains of NSCDs [50,51]. On the other hand, the PL band peaked at 564 nm is typical of OPD_ox_ [23,24,25,26]. In fact, PL excitation (PLE) spectra in Figure 3b shows that this visible emission is most excited in the range around 450 nm, where the absorption of OPD_ox_ occurs. However, it is still slightly excited in the UV range, possibly due to some energy transfer mechanism from within the NSCDs matrix or from direct OPD_ox_ absorption at this wavelength, even though to a much lesser extent.

### 3.3. Results–Colorimetric and Fluorescence Sensing Properties

#### 3.3.1. Absorption-based Sensing

**Figure 4:**
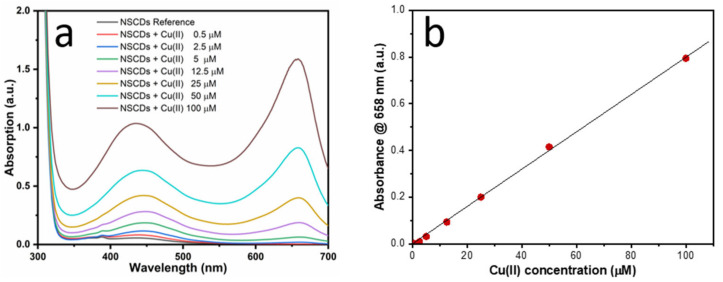
(**a**) UV–Vis absorption spectra of NSCDs-sensing solution upon the addition of Cu(II) ions at different concentrations; (**b**) calibration curve.

Our previous study [44] demonstrated that absorption-based sensing of Cu(II) can be achieved through NSCDs due to the formation of cuprammonium complexes with consequent arising of a peculiar absorption band at 660 nm (see Figure 4a), a region where pure NSCDs are completely transparent. This gives rise to a distinct visual color change from light yellow to deep green (see Figure S2). In addition, a minor shoulder arising at 560 nm in the presence of cobalt produced a different color change that did not interfere with response to copper [44]. In fact, absorbance at 660 nm in the presence of Cu(II) showed good linearity vs. ion concentration, as displayed by Figure 4b, enabling a remarkable value of LOD = 100 nM with very low sensitivity to other potentially interfering HM ions (Figure S3). Addition of copper also induced a shift of ζ-potential to around 25 mV (Figure S1b), indicating that Cu(II) ions are bound to the surface of NSCDs and produce a positively charged surface on the particles.

On the other hand, it was also evident that the absorption band at 430 nm increased with copper concentration, probably as a consequence of further oxidation of OPD to OPD_ox_ promoted by Cu(II) ions. As mentioned in the Introduction, this effect can also occur in pure (unbound) OPD and is generally achieved with the help of some catalyzer. In fact, we found that Cu(II) ions took several hours to give rise to a significant absorption band at 430 nm in a water solution of OPD in the absence of NSCDs (see Figure S4). In contrast, the same effect occurred in less than 5 min in the presence of NSCDs. Oxidation of the OPD solution also happened by simple exposure to air, but it was only visible at much higher concentrations. The production of more OPD_ox_ by Cu(II) is reflected in the fluorescence properties of NSCDs, as it has been further investigated for application to sensing and described below.

#### 3.3.2. Fluorescence-based Sensing

**Figure 5:**
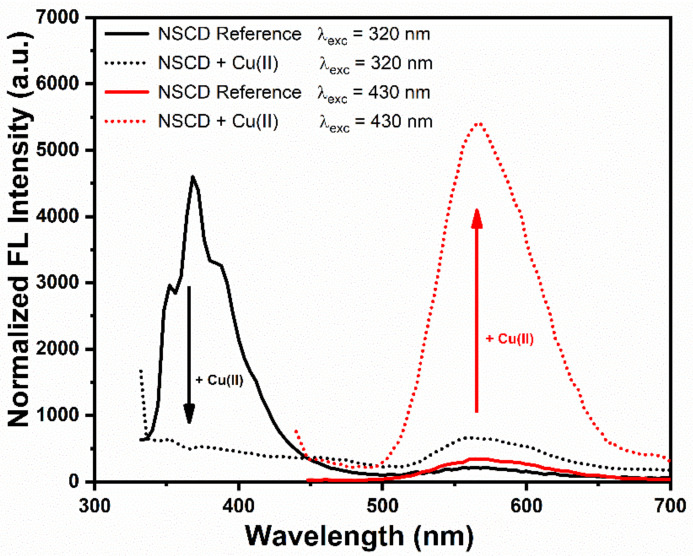
Variation of fluorescence emission of NSCD sensing solution excited at 320 nm (black curve) or 430 nm (red) upon the addition of 100 µM Cu(II).

Figure 5 shows that addition of Cu(II) produced two different simultaneous effects on fluorescence of NSCDs. Indeed, the UV band at 370 nm was strongly quenched while the visible emission at 564 nm was significantly enhanced.

Fluorescence quenching is quite common in CDs interacting with metal ions [34,35] and is usually attributed to electron-transfer mechanisms from carbon nanomaterials, with delocalized π-electron systems, towards electron-withdrawing agents such as transition metal ions with partly filled d orbitals. In fact, we observed that fluorescence quenching at 370 nm showed very low selectivity towards the different 10 metal ions tested in the present study (not reported here).

**Figure 6:**
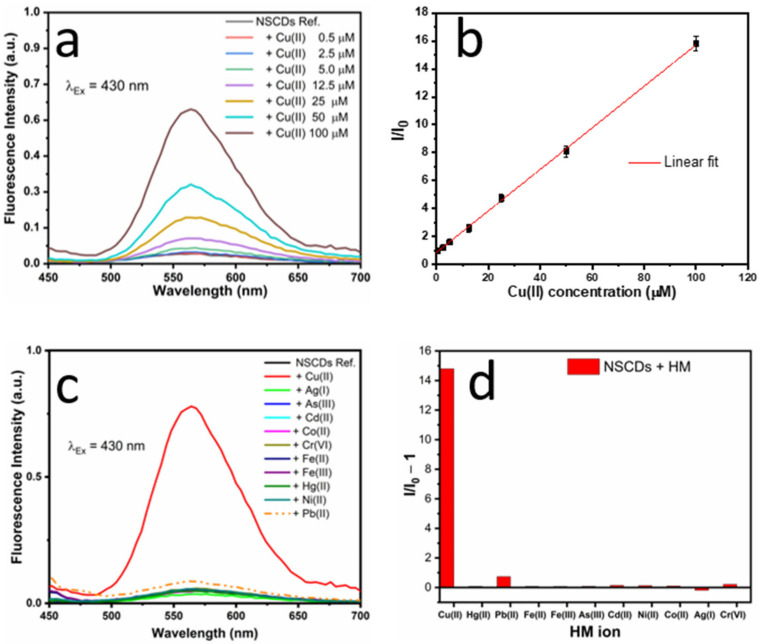
(**a**) Emission spectra of NSCDs in the presence of Cu(II) at different concentrations. (**b**) calibration plot showing the fluorescence enhancement intensity vs. the Cu(II) concentration over the range 0–100 μM; (**c**) emission spectra of NSCDs in the presence of different metal ions at a concentration of 100 µM; (**d**) selectivity of fluorescence response of NSCDs.

On the other hand, the enhancement of fluorescence at 564 nm demonstrated very good linearity with Cu(II) concentration in the range 0.5–100 µM and excellent selectivity, as shown in Figure 6. In particular, no response to cobalt was found, in contrast to the case of absorption. We believe that the fluorescence enhancement, which paralleled the increase in absorbance at 430 nm (see Figure 4), is produced by further oxidation of residual OPD, which is converted into OPD_ox_ by the presence of Cu(II). In order to investigate this hypothesis, we recorded the fluorescence emission spectra of pure OPD solutions after oxidation, either achieved by addition of Cu(II) or by simple exposure to air and compared them with the emission of NSCDs both in the presence and in the absence of Cu(II), as reported in Figure S5. All the fluorescence spectra were very similar and showed similar values of fluorescence lifetime (See Table S1 in Supplementary Materials) thus supporting the common nature of the emission processes. In particular, the fact that the lifetime of the emission of NSCDs does not vary significantly in the presence of Cu(II) indicates the occurrence of a static enhancement, in agreement with the hypothesis of cuprammonium complex formation. Further studies, however, are needed to provide an exhaustive explanation of the physical/chemical origin of the fluorescence enhancement.

It should be noted that the fluorescence enhancement was easily visible by naked eye and, remarkably, also appeared under green light excitation, which can be provided by more available and cheap light sources (Figure S6). Interestingly, there was no significant response to Ag(I), whereas OPD is known to be easily oxidized by the presence of silver ions as well [23,26]. That is probably due to the poor stability of silver in basic solutions and its strong affinity with sulfur, which could bind Ag(I) ions to the sulfur-containing groups of NSCDs thus preventing effective interactions with OPD.

The linear calibration obtained by plotting the ratio I/I_0_ over the concentration of copper (II) enabled the estimate of LOD = 1 µM for Cu(II) according to Equation (1):

LOD = 3 σ/m (1)

where σ is the standard deviation (n = 3) of the reference fluorescence data and m is the slope of the linear calibration curve, as per IUPAC guidelines [52]. As for the absorption-based sensing, the calculated LOD for the fluorescence method is much lower than the limits set by either WHO ~30 μM (2 mg/L) or the EPA ~20 μM (1.3 mg/L).

### 3.4. Comparison with Other Sensing Systems and Techniques

Other techniques and nanomaterials that have recently been investigated for detection of Cu(II) are reported in Table 1, together with their respective LODs and linearity ranges, in comparison with the present system. The LOD of the present method (0.1 µM and 1 µM for colorimetric and fluorometric response, respectively) is outperformed by most of the approaches reported, both based on CDs [30,53,54] and other nanomaterials, e.g., glutathione-modified quantum dot (GSH-CdTe QDs) [55]. However, it should be noted that the LOD of the NSCD solution is still much lower than the guideline value set by WHO (30 µM). On the other hand, its linearity range is broader than that of all of the other methods considered here. More importantly, most other techniques rely on quite complex and time-consuming sensing procedures. For example, the method described by Tabakci B. and collaborators [56], which, however, presented a significantly higher LOD (5.1 µM and 0.96 µM) and a moderate linearity range, used the Cu(II)-picolylamine-armed calix [4]arene (4-PACX) complex formation and also included non-friendly solvents. The fluorescence detection of copper with OPD-derived carbon dots [30] required that the stock solutions of the Cu(II) ions were first diluted with 10 mM of phosphate-buffered saline (PBS) at pH 7.0 and then mixed with 10 mM PBS (pH 7.0) containing C dots for incubation at 37 °C for 1 h. Similarly, the remarkable LOD of 10 nM obtained with GSH-CdTe QDs [55] required semiconductor-based nanomaterials, which present high toxicity, intrinsic blinking, chemical instability, and insolubility in aqueous solution. In contrast, the present sensing experiment required only the addition of the water sample to the sensing solution in a 1:1 volume ratio, and the result could be obtained in 10 min at room temperature without the needs of pH buffered solutions or any other procedure.

### 3.5. Stability and Reproducibility of NSCD Synthesis

We have observed that OPD oxidation also occurs at a certain speed after the synthesis of NSCDs by simple exposure to air, calling for some precaution in handling the material. As reported in Figure S7, if the as-prepared NSCD mother solution is sealed and stored at 4 °C and diluted to the concentration for sensing not earlier than a day before the sensing experiment, quite accurate results can still be obtained after 3 months, with regard to the absorption-based colorimetric sensing. Figures S8 and S9 (which summarizes both Abs. and Fluor. results) show that the situation is more critical for the fluorescence enhancement since accurate results can only be obtained within 10 days after synthesis. In fact, present fluorescence results clearly make the system hardly suitable for application as is and further studies are needed to investigate a chance of improving stability performance.

On the other hand, as displayed by Figure S10, both the absorption and fluorescence enhancement effects show variations within ±15% over different synthesis batches, which we believe quite acceptable for a laboratory preliminary study.

### 3.6. Results—Colorimetric and Fluorescent Sensing with Solid-State Agarose Matrices

To further explore the applicability of the present system, we investigated the possibility of incorporating NSCDs in solid-state matrices, in place of liquid dispersion, and thus prepared agarose-based NSCDs (*Aga-NSCDs*) thick films as described in Section 2.4. This strategy could permit easier testing procedures by simple immersion of the sensing material in water samples, even in a continuous mode, for simple visual response.

**Figure 7:**
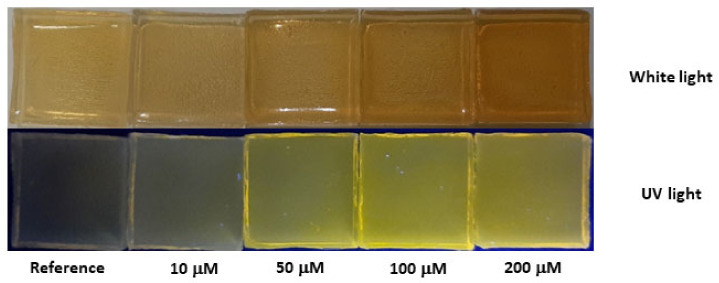
Photographs of colorimetric sensing test with hydrogel *Aga-NSCD* films after immersion for 2 h in a DI water at different Cu(II) concentrations.

Figure 7 displays two photographs of five similar *Aga-NSCD* films after a 2-h soaking in water samples with different Cu(II) concentrations. Either white daylight on a black background or 365-nm UV light on a white one was used for the illumination of samples.

**Figure 8:**
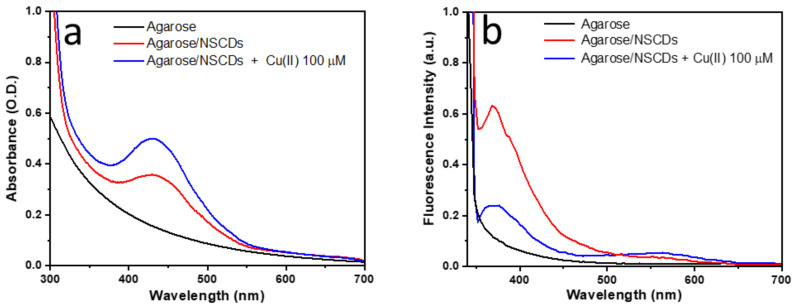
(**a**) UV-vis absorption and (**b**) fluorescence emission spectra of hydrogel *Aga-NSCD* films after immersion in DI water with a Cu(II) concentration of 100 µM. Emission spectra were acquired with l_exc_ = 320 nm.

It appears that concentrations as low as 10 μM can easily be detected both in daylight and especially under UV excitation due to change of the perceived color. In order to understand the spectroscopic origin of this color change, absorption and fluorescence measurements were performed on the *Aga-NSCD* films as shown in Figure 8. The UV-Vis absorption spectrum of *Aga-NSCD* (Figure 8a, red curve) can be considered as the superposition of the spectra of the two components. In particular, the absorption band of OPD_ox_ at 430 nm is clearly observed over the absorbance tail of agarose. This band is enhanced after interaction with copper (blue curve) as a consequence of further oxidation of OPD by Cu(II) ions. Interestingly, the interaction with copper did not produce a rising absorption band at 660 nm as in water dispersion, suggesting that the formation of cuprammonium complexes is strongly hampered by the agarose matrix. Similar to absorption, the fluorescence spectrum of *Aga-NSCD* (Figure 8b, black curve) resembles the superposition of the spectra of the two components, with the emission of OPD_ox_ at 564 nm not significantly affected by interaction with the solid matrix. Interaction with Cu(II) quenched the fluorescent UV/blue emission at 370 nm, even though to a lower degree than in liquid, and slightly enhanced the emission of OPD_ox_ at 564 nm.

**Figure 9:**
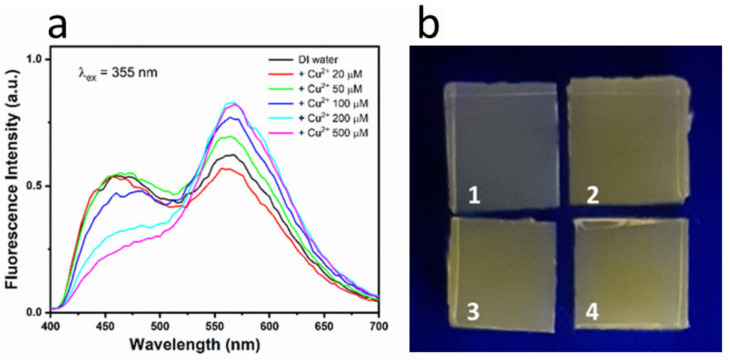
(**a**) Fluorescence emission spectra of hydrogel *Aga-NSCD* films after immersion in DI water with different Cu(II) concentration; (**b**) photographs of colorimetric sensing with hydrogel *Aga-NSCD* films (1) in pure DI water, 100 µM Cu(II) in (2) DI water, (3) tap water and (4) lake water.

To simulate the visual effect for the eye under blacklight (UV-lamp), fluorescence spectra were taken by using a 355-nm excitation and a 400-nm cut-off filter on the emission signal, as shown in Figure 9a. This makes more evident the progressive decrease in the blue emission along with the increase in yellowish fluorescence at 564 nm due to further oxidation of OPD as the concentration of Cu(II) ions is increased. The dependence of the visible emission on the copper concentration can account for the color change perceived in solid-state matrices.

Figure 9b shows the colorimetric sensing with *Aga-NSCD* in real water samples. In particular, we prepared spiked samples of tap water of the city aqueduct of Rome (east area) and of lake water taken from Lago Albano, a volcanic lake 30 km south of Rome. The real water samples were simply filtered through a 0.2 μm filter and spiked with 10 μL of DI copper solution to reach the final ion concentration.

Specifically, in Figure 9b, the reference sample in pure DI water (1) is compared with similar samples sensing 100 µM Cu(II) in (2) DI water, (3) tap water and (4) lake water. Even though no quantitative measurements were performed, the visual observation confirmed that no significant variation of the color change was observed in real water samples in comparison with the DI water one. We believe that these qualitative results are quite promising for the real practicability of this method, especially if one considers the high permanent hardness of the drinking water in Rome (33 fH), mostly due to a calcium content as high as 101 mg/L (2.5 mM).

Finally, Figure S11 shows that selectivity to Cu(II) was still effective in *Aga-NSCD* with the exception of Co(II), due to the absorbance response to this ion already observed in liquid solutions (see Section 3.3.1 and Figure S2). The simultaneous sensitivity of hydrogel *Aga-NSCD* films to Co(II), however, can be distinguished from that to Cu(II) through a comparison with the fluorescence response of the liquid NSCD solution, which is only sensitive to copper. In other words, in principle, a logic gate behavior could be exploited to detect and distinguish between the presence, even simultaneously, of Cu(II) and Co(II).

## 4. Conclusions

The nitrogen and sulfur co-doped NSCDs prepared by using a simple one-pot hydrothermal method demonstrated selective optical responses to Cu(II) in water, both through naked-eye color changes and simultaneous enhancement of visible fluorescent emissions. Specifically, a rising absorption band at 660 nm can be explained by formation of cuprammonium complexes through coordination of copper ions with amino functional groups of NSCD. This is consistent with the shift of ζ -potential from close to 0, in pure NSCDs, to around 25 mV in the presence of Cu(II) and indicates that the copper ions are bound to the surface of NSCDs and produce a positively charged surface. In contrast, the spectral profile, excitation spectrum and lifetime of the fluorescent band at 564 nm strongly support the hypothesis that this emission comes from OPD bound to NSCDs that is further oxidized in the presence of Cu(II). Both absorption and fluorescence variations of NSCDs showed a linear dependence on copper concentration in the range 1–100 µM, even in the presence of other metal cations, with the lowest detection limit of 100 nM and 1 µM, respectively. However, fluorescence results showed low stability with time since the prepared NSCDs showed a decreasing fluorescence enhancement effect after one week from preparation. This clearly makes the system hardly suitable for application as is and further studies are needed to improve stability performance.

Incorporation of NSCDs in a hydrogel agarose matrix for application to sensing was successfully achieved, but at the cost of the absorbance at 660 nm, which was completely suppressed. Clearly, the agarose matrix interacts with the amine groups and strongly hampers the formation of cuprammonium complexes. However, oxidation of OPD was still effective and produced perceivable color variations both in fluorescent emission, even under visible-light excitation, and daylight vision. These color changes were similarly perceived in tap and lake water samples allowing for the visual detection of Cu(II) in the medium-micromolar concentration range. The present material could thus be used both in the form of liquid and solid matrix, respectively, with different colorimetric responses to the same analyte in view of improved selectivity and reliability. Further optimization of the present method might be useful for simple, cost-effective, visual monitoring of copper onsite, for instance in mining and industrial wastewater samples.

## Figures and Tables

**Table 1:** Comparison of the performances of various optical techniques and nanomaterials for Cu(II).

Materials	Method	Linear Range (µM)	Limit of Detection (µM)	Reference
Receptor L	Colorimetric	0–50	2.82	[57]
Rhodamine	Colorimetric	0–30	0.48	[58]
Dibenzo[b,j][1,10]Phenanthroline	Colorimetric	10–100	0.14	[59]
picolylamine-armed calix [4]arene (4-PACX)	Colorimetric Fluorescence	1–11 1–50	5.1 0.96	[56]
GSH-CdTe QDs	Fluorescence	0.02–1.1	0.0101	[55]
CDs	Colorimetric Fluorescence	0.01–10 0.1–2	0.004 0.09	[53]
CDs	Fluorescence	0.5–7	0.15	[60]
N-CDs	Fluorescence	0.05–25	0.023	[61]
Adenine-stabilized CDs	Fluorescence	0.001–0.75	0.0003	[54]
CDs	Fluorescence	0.002–0.080	0.0018	[30]
NSCDs	Colorimetric Fluorescence	1–100 0.5–100	0.1 1	Present study

## Supplementary Materials

The following are available online at https://www.mdpi.com/article/10.3390/s23063029/s1, Figure S1: Zeta potential of NSCDs sensing solution: (a) with no Cu^2+^; and (b) with 100 µM Cu^2+^. Table S1: Fluorescence lifetime @564 nm. Figure S2: Selective visual response of NSCDs sensing solution under white light upon the addition of different HM ions at a concentration of 100 µM. Figure S3: Interference from other HMs (blue bars) in the NSCDs sensing solution. Figure S4: Absorbance spectra of OPD at a concentration of 100 µM in DI water (black curve), the OPD solution immediately after the addition of 100 µM Cu(II) (blue), after 1 h (orange) and after 16 h at room temperature (red). Figure S5: Fluorescence emission spectra of NSCD sensing solution (black curve), NSCD sensing solution 10 min after the addition of 100 µM Cu(II) (red), OPD at a concentration of 100 µM in DI water 16 h after the addition of 100 µM Cu(II) (green), OPD at a concentration of 20 mM in DI water 16 h after exposure to air. All the spectra are excited at l_exc_ = 430 nm and normalized to the intensity at 564 nm. Figure S6: Fluorescence of NSCD sensing solution under different light excitations upon the addition of different HM ions at a concentration of 100 µM. Figure S7: Long-term stability of the absorption spectrum of the NSCD sensing solution stored as concentrated mother solution and prepared by dilution immediately before the sensing experiment. Figure S8: Long-term stability of the fluorescence spectrum of the NSCD sensing solution stored as concentrated mother solution and prepared by dilution immediately before the sensing experiment. Figure S9: Long-term stability of the absorption variation and the fluorescence enhancement of the NSCD sensing solution. Both the quantities are normalized to the respective initial values. Figure S10: Reproducibility of the absorption variation and the fluorescence enhancement of the NSCD sensing solutions prepared on different days. Figure S11: Selectivity of sensing with hydrogel *Aga-NSCD* films. Photographs of hydrogel *Aga-NSCD* films after immersion in a DI water solutions of different HMs.
