# Supplementary material for: Colorimetric and Fluorescent Sensing of Copper Ions in Water through o-Phenylenediamine-Derived Carbon Dots
Source: Sensors (Basel). 2023 Mar 10;23(6):3029. doi: 10.3390/s23063029 (PMC10056730; doi:10.3390/s23063029)
Supplement: Supplementary file 1 [file sensors-23-03029-s001.zip › sensors-2249416-supplementary.pdf]

sensors

MDPI

Supplementary Materials

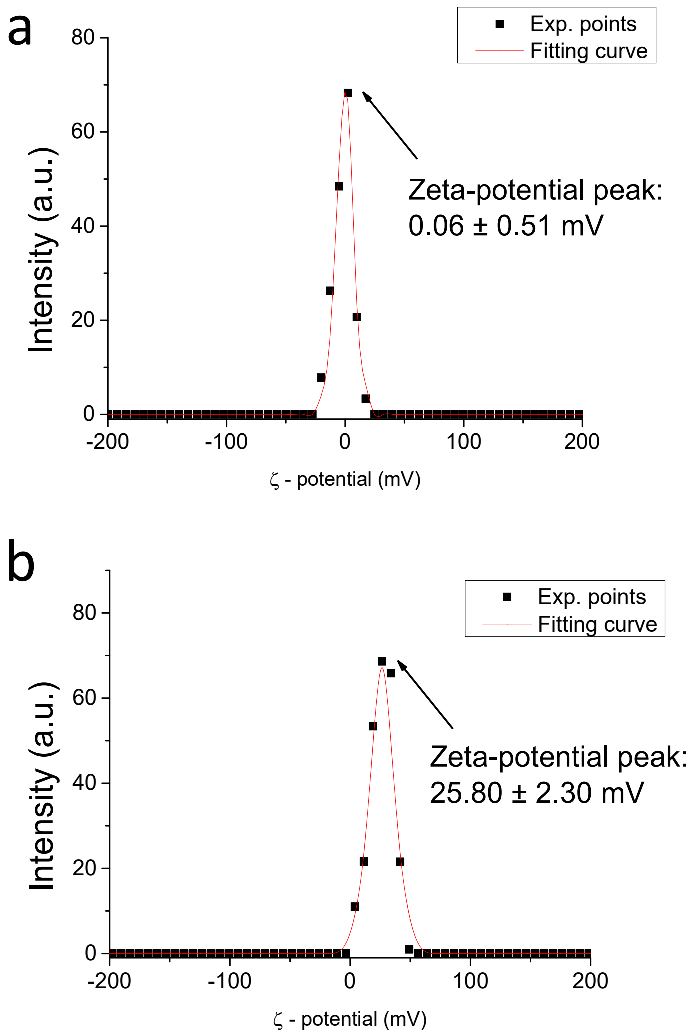

Figure S1. Zeta potential of NSCDs sensing solution: (a) with no Cu^{2+}; and (b) with 100 μM Cu^{2+}.

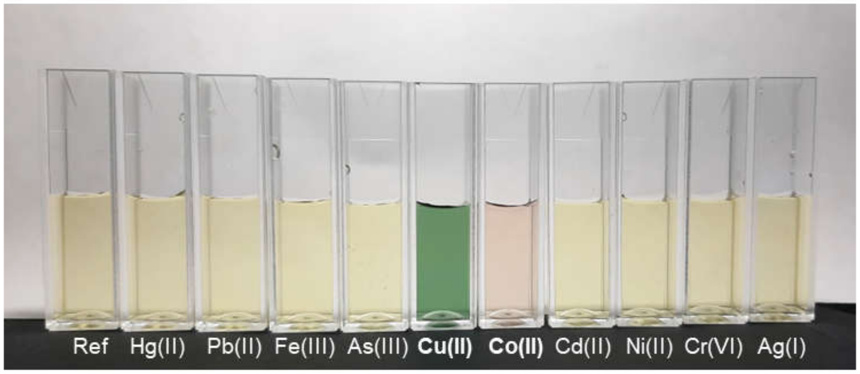

Figure S2. Selective visual response of NSCDs sensing solution under white light upon the addition of different HM ions at a concentration of 100 μM.

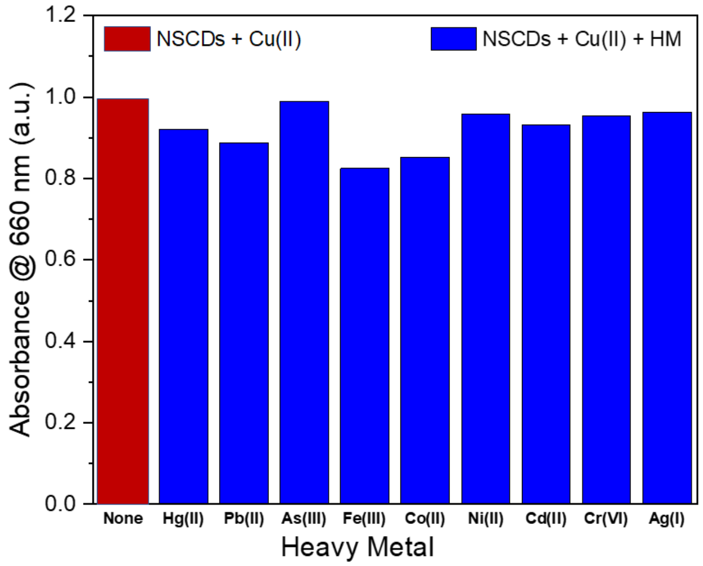

Figure S3. Interference from other HMs (blue bars) in the NSCDs sensing solution.

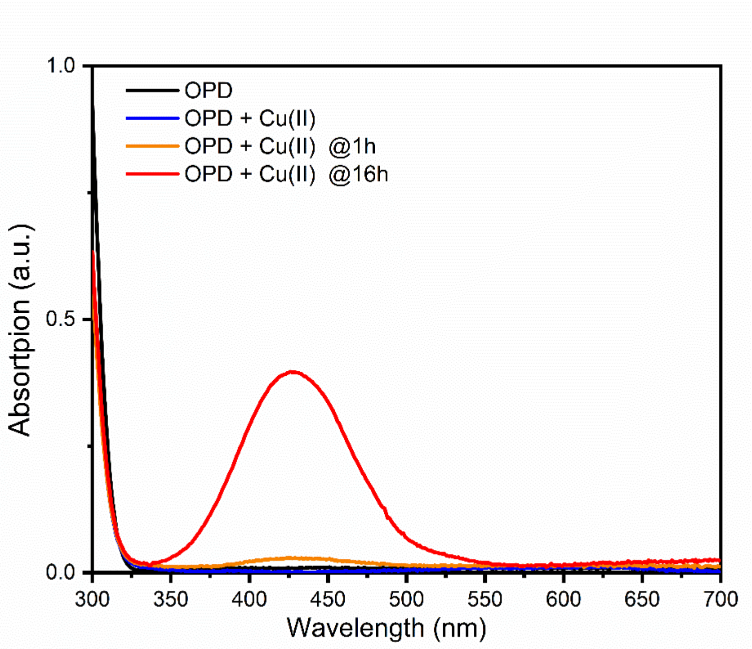

Figure S4. Absorbance spectra of OPD at a concentration of 100 µM in DI water (black curve), the OPD solution immediately after the addition of 100 µM Cu(II) (blue), after 1 h (orange) and after 16 h at room temperature (red).

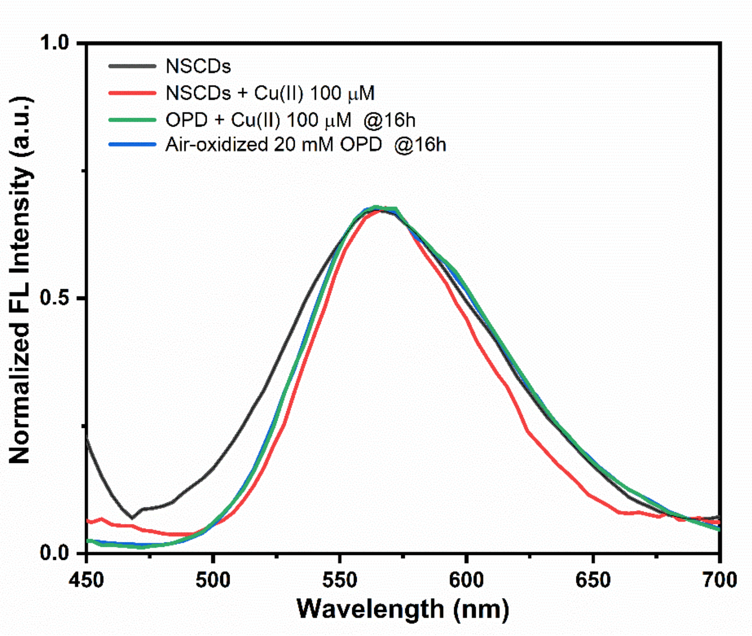

Figure S5. Fluorescence emission spectra of NSCD sensing solution (black curve), NSCD sensing solution 10 minute after the addition of 100 μM Cu(II) (red), OPD at a concentration of 100 μM in DI water 16 hours after the addition of 100 μM Cu(II) (green), OPD at a concentration of 20 mM in DI water 16 hours after exposure to air. All the spectra are excited at l_{exc} = 430 nm and normalized to the intensity at 564 nm.

Table S1. Fluorescence lifetime @564 nm.

Samples	Lifetime (τ) ns	X²
NSCDs	1.83	6.24
NSCDs + Cu(II)	1.76	8.65
OPD + Cu(II)	1.55	9.42
Air-oxidized OPD	1.4	7.34

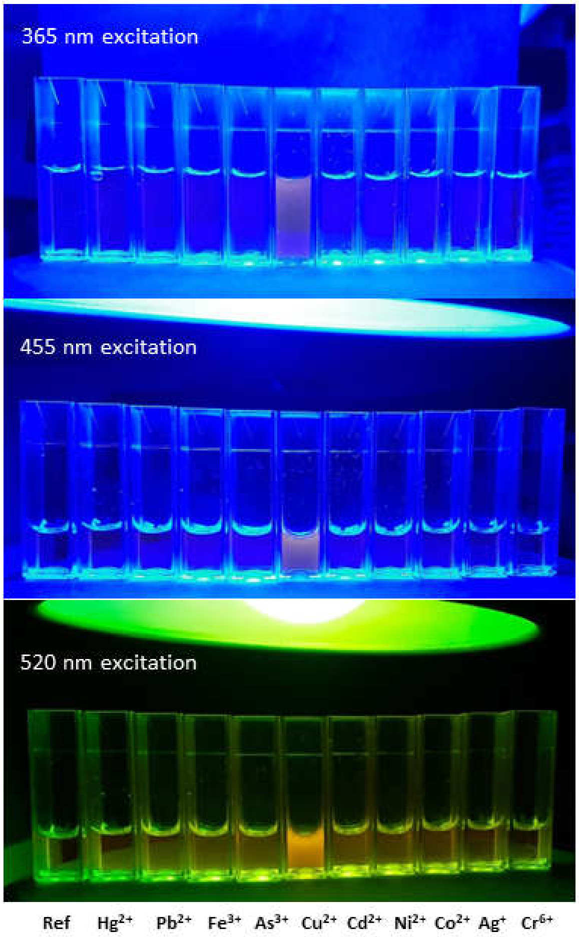

Figure S6. Fluorescence of NSCD sensing solution under different light excitations upon the addition of different HM ions at a concentration of 100 μM.

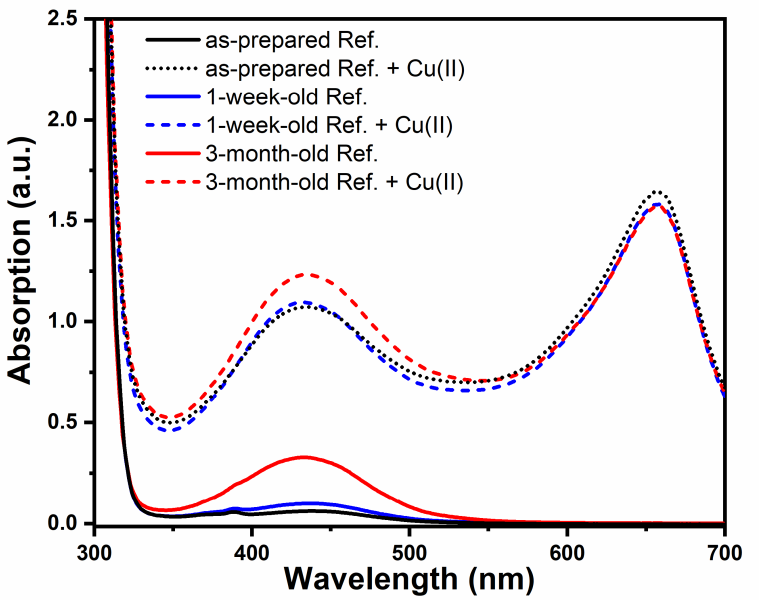

Figure S7. Long-term stability of the absorption spectrum of the NSCD sensing solution stored as concentrated mother solution and prepared by dilution immediately before the sensing experiment.

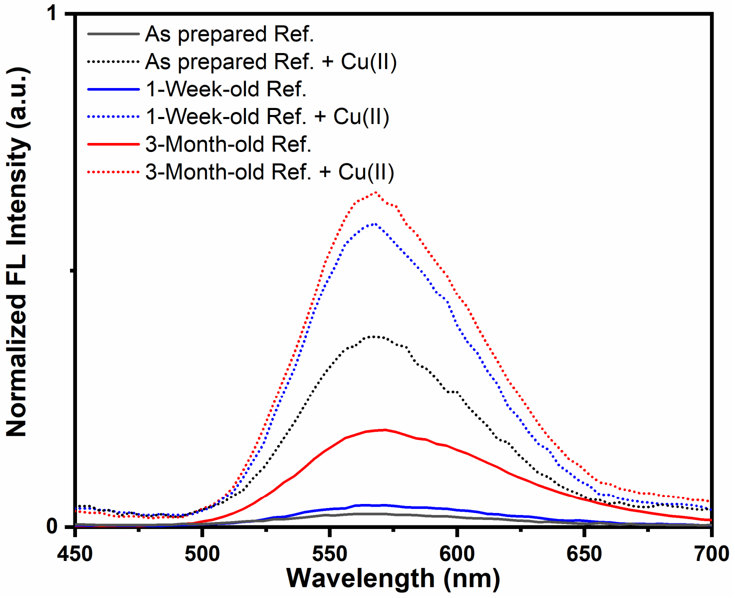

Figure S8. Long-term stability of the fluorescence spectrum of the NSCD sensing solution stored as concentrated mother solution and prepared by dilution immediately before the sensing experiment.

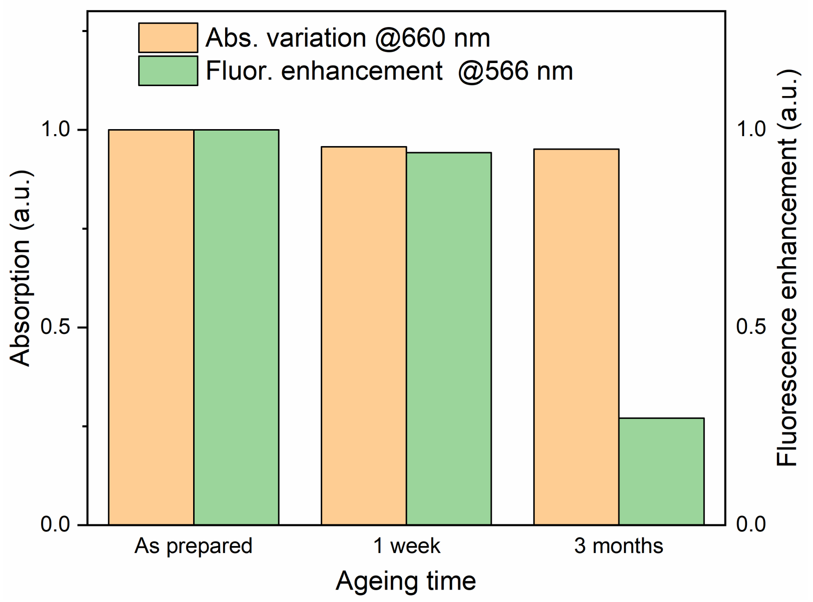

Figure S9. Long-term stability of the absorption variation and the fluorescence enhancement of the NSCD sensing solution. Both the quantities are normalized to the respective initial values.

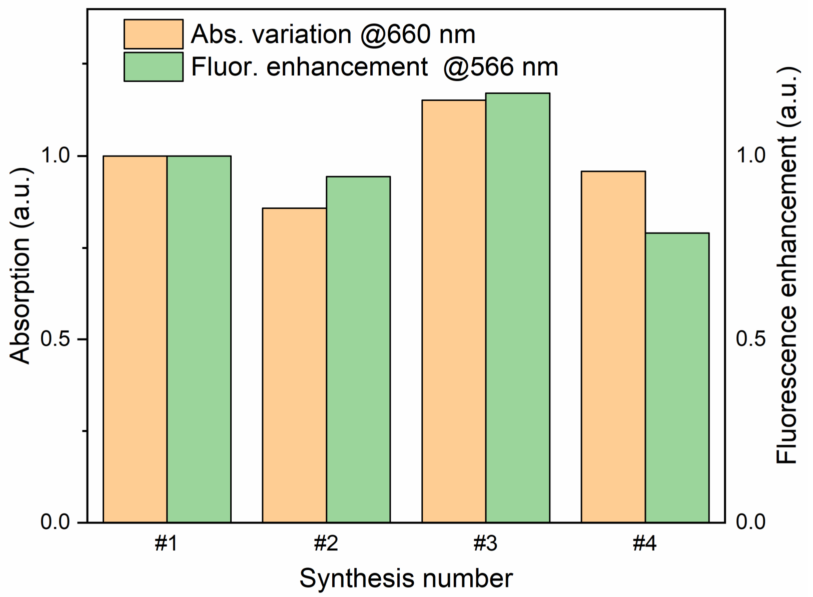

Figure S10. Reproducibility of the absorption variation and the fluorescence enhancement of the NSCD sensing solutions prepared on different days.

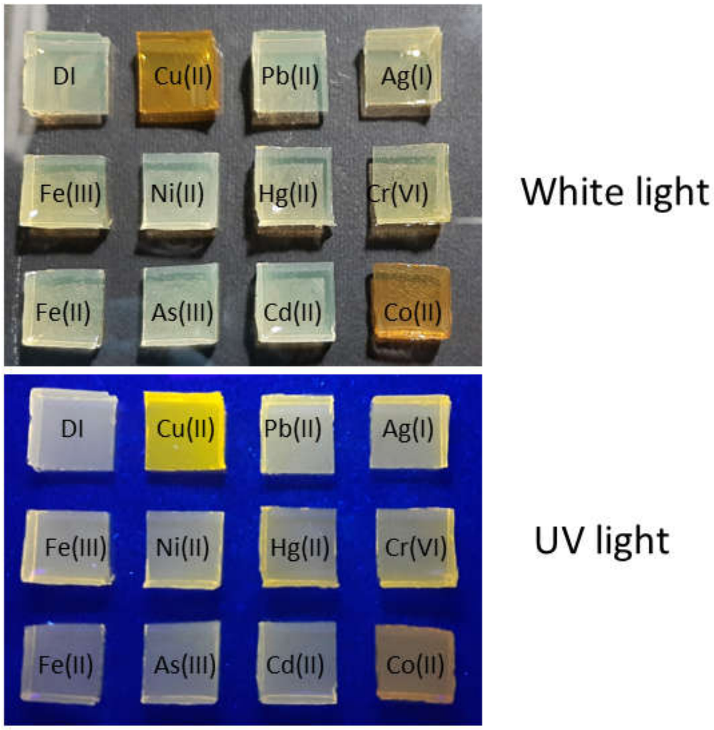

Figure S11. Selectivity of sensing with hydrogel Aga-NSCD films. Photographs of hydrogel Aga-NSCD films after immersion in a DI water solutions of different HMs.
